# Accurate and Low-Cost Cardiac Disorder Detection from Wearable Phonocardiogram Signals Using Hybrid Feature Selection and Machine Learning

**DOI:** 10.3390/bios16060310

**Published:** 2026-05-29

**Authors:** Ali Narin, Rukiye Uzun Arslan, Damla Kırkıl

**Affiliations:** Department of Electrical and Electronics Engineering, Zonguldak Bülent Ecevit University, Zonguldak 67100, Türkiye; rukiye.uzun@beun.edu.tr (R.U.A.); d.kirkil@beun.edu.tr (D.K.)

**Keywords:** phonocardiogram, feature selection, hybrid optimization, intelligent stethoscope, computer-aided diagnosis

## Abstract

Early and reliable identification of cardiac disorders from Phonocardiogram (PCG) signals acquired from wearable biosensors is critical to support clinical decision making and reduce subjectivity in auscultation-based assessments. This study proposes a multi-stage hybrid feature selection-classification approach to increase diagnostic accuracy without requiring computationally expensive deep learning (DL) architectures. First, the most statistically discriminative features were identified using mRMR, ReliefF, and Kruskal–Wallis filtering methods. Particle swarm optimization (PSO) and ant colony optimization (ACO) were then applied to optimize the solution space. Finally, the selected feature subsets were tested with k-nearest neighbor (k-NN), support vector machines (SVMs), and Bagged Tree (BT) classifiers. Experimental results show that the proposed method significantly increases the model robustness and generalizability. In particular, the Kruskal–Wallis+k-NN and ReliefF+k-NN combinations achieved competitive performance compared to many DL-based approaches in the literature, with 99.80% accuracy and 99.50% F1-score. Furthermore, hybrid models augmented with PSO and ACO also achieved 99.60% accuracy. The findings demonstrate that well-designed feature selection strategies offer high accuracy and enhanced clinical applicability while using only a small set of handcrafted features and conventional classifiers. Therefore, the proposed framework is a strong candidate for smart stethoscope-based early screening solutions.

## 1. Introduction

Cardiovascular diseases (CVDs) remain the foremost cause of mortality worldwide, posing a persistent challenge to global health systems. According to the World Health Organization (WHO), these diseases are responsible for millions of deaths each year, with approximately 20 million fatalities reported in 2022 alone—corresponding to 31% of all global deaths [1]. A similar pattern is observed at the national level; data published by the Turkish Statistical Institute (TUIK) show that circulatory system diseases accounted for 36.8% of all deaths in Turkey in 2023, making them the leading cause of mortality in the country [2]. CVDs encompass a wide range of disorders, including congenital heart disease, arrhythmias, coronary artery disease, myocardial infarction, heart failure, and rheumatic heart disease [3]. Despite their clinical diversity, these conditions share a common consequence: disruption of the normal structure and function of the heart. Such alterations often progress insidiously and may result in serious complications if not recognized in time. Therefore, achieving early and accurate diagnosis is of critical importance for improving patient prognosis and reducing mortality rates [4].

In routine clinical settings, commonly used diagnostic methods are effective but not always practical. Many of these techniques involve high costs, prolonged evaluation times, and a strong dependence on specialist expertise. Such limitations can restrict their widespread use and highlight the need for alternative approaches that are simpler, more accessible, and cost-efficient [5]. In this context, phonocardiography (PCG) provides a practical and non-invasive means of capturing heart sounds, which contain important clues about cardiac function [6].

PCG is particularly relevant for detecting valvular heart diseases (VHDs), including aortic stenosis (AS), mitral stenosis (MS), mitral regurgitation (MR), and mitral valve prolapse (MVP). These disorders arise due to abnormalities in the structure or function of heart valves, resulting either in limited opening or incomplete closure [7]. For example, AS restricts blood flow from the left ventricle as a result of valve narrowing, while MS reduces the transfer of blood between the left atrium and left ventricle. In the case of MR, blood flows backward due to improper valve closure, whereas MVP is characterized by the abnormal movement of the valve into the left atrium during systole. These conditions produce distinct acoustic patterns, which can be observed in PCG recordings and used for diagnostic purposes.

However, the manual interpretation of PCG signals is not without challenges. It is both time-intensive and subject to variability between clinicians, which can affect diagnostic consistency. To address these issues, automated systems based on machine learning (ML) have been increasingly investigated. These systems enable faster and more objective analysis of PCG signals, while also offering advantages in terms of consistency and reproducibility.

Recent studies have explored the use of both traditional ML and deep learning (DL) approaches in this area [8,9,10,11,12]. Much of the studies have focused on improving different stages of the analysis process, including preprocessing, feature extraction, and feature selection, often accompanied by comparisons between classification models. Preprocessing is typically applied to enhance signal quality through operations such as noise reduction, filtering, segmentation, and normalization [13,14,15,16,17,18,19]. In the feature extraction stage, methods based on time, frequency, and time–frequency domains—such as Mel-Frequency Cepstral Coefficients (MFCCs) and wavelet transforms—are commonly used to obtain meaningful representations of heart sounds [20,21,22,23]. For classification, algorithms such as Support Vector Machines (SVM), Random Forests (RF), and k-Nearest Neighbors (k-NN) remain widely used due to their stable performance and relative interpretability across different datasets [24,25,26,27,28,29,30,31]. Conversely, DL-based models such as Convolutional Neural Networks (CNNs) and Long Short-Term Memory (LSTM) networks have achieved remarkable accuracy by capturing the spatial and temporal dependencies within PCG signals [15,16,19,20,21,23,32,33,34,35,36,37,38].

### Motivation and Innovations

PCG signals contain critical physiological information reflecting the mechanical functions of the heart, and the automatic classification of pathological heart sounds is becoming increasingly important in the development of non-invasive diagnostic support systems. Despite numerous studies in the literature, several fundamental limitations persist. First, most existing studies focus on a single feature domain, limiting comprehensive analyses using all time, time-frequency, and nonlinear features. Second, feature selection is typically performed solely using filter-based or meta-heuristic methods; however, both methods have their strengths and weaknesses, with the single approach often resulting in a redundant feature set, over fitting, and limited generalization performance. Furthermore, the literature reveals that the classification performance of different cardiac pathologies is uneven and that most studies do not provide generalizable diagnostic models suitable for clinical application.

**Figure 1:**
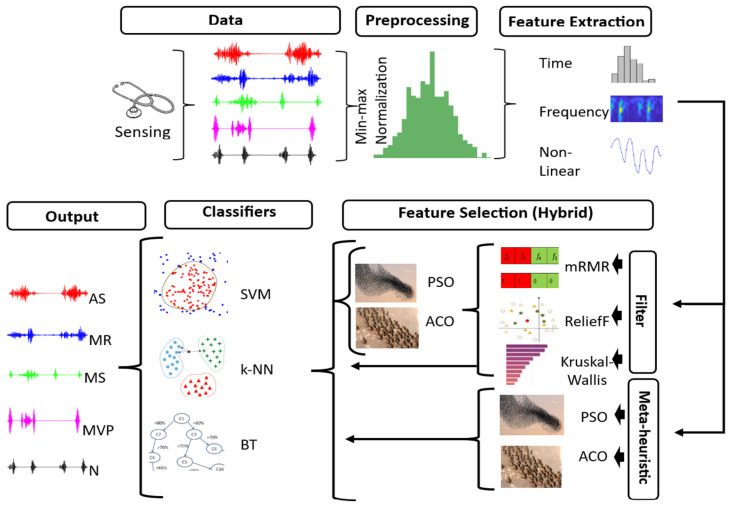
Schematic representation of the proposed model.

Based on these shortcomings, this study proposes a comprehensive classification framework based on PCG signals, detailed in Figure 1. The proposed approach utilizes (i) min–max normalization, (ii) multi-domain (time, time–frequency, and nonlinear) feature extraction, and (iii) systematic comparison of the full feature set, filter-based selection, meta-heuristic selection, and hybrid selection methods with SVM, k-NN, and Bagged Trees (BT) classifiers. The goal is to maximize diagnostic accuracy, reduce model complexity, and increase generalization capacity for five cardiac classes.

The main scientific contributions of this study are summarized below:

- An integrated PCG classification pipeline based on multi-domain feature representations, including time-domain, frequency-domain, and nonlinear features, was proposed.
- A four-stage feature evaluation strategy was developed, enabling a systematic comparison of all features, filter-based methods, meta-heuristic approaches, and hybrid feature selection techniques using the same dataset.
- A novel hybrid feature selection framework combining the minimum redundancy maximum relevance (mRMR), ReliefF, Kruskal–Wallis, Ant Colony Optimization (ACO), and Particle Swarm Optimization (PSO) was proposed, yielding a notable improvement in class discrimination performance.
- A comprehensive comparative analysis was conducted using SVM, k-NN, and BT classifiers, and the models achieving the highest classification performance were identified.
- High generalization performance was achieved across five cardiac conditions, namely AS, MR, MS, MVP, and Normal (N), highlighting the potential applicability of the proposed approach in clinical decision support systems.

## 2. Material and Methods

The methodological framework adopted in this study was designed as a systematic pipeline for the classification of PCG signals. The workflow comprises the following stages: (i) dataset preparation, (ii) feature extraction to derive meaningful parameters from the signals, (iii) dimensionality reduction and optimization of the selected features using PSO, (iv) model training of various ML models, and (v) performance evaluation of the developed models using multiple statistical metrics.

### 2.1. Data Set

The dataset utilized in this study was retrieved from GitHub (https://github.com), a widely used open-source software development and data-sharing platform. It comprises five clinically significant cardiac pathology classes that play a crucial role in cardiovascular disease diagnosis: AS, MR, MS, MVP, and N cardiac activities [24,39].

The composition of the dataset consists of an equal number (n = 200) of PCG recordings for each pathology class, with a total of 1000 heart sound recordings analyzed. This balanced class distribution was specifically chosen to minimize the risk of algorithmic bias arising from class imbalance during the training of ML models [40,41]. It is widely accepted in the literature that balanced datasets are critical for the objective evaluation of classification performance and for improving the generalization capacity of models [42].

Data preprocessing was performed in three main stages in accordance with widely accepted signal processing protocols. In the first stage, the original sampling frequency of 8 kHz was retained for all PCG recordings to preserve signal quality and temporal resolution. This sampling frequency was selected based on the criteria of fully covering the frequency spectrum of heart sounds (0.5–2.5 kHz) and meeting the requirements of the Nyquist-Shannon sampling theorem [43].

In the second stage, the min–max normalization technique was applied to standardize signal amplitudes. This normalization method eliminates amplitude variations arising from different recording conditions and scales the signals to the [−1, 1] dynamic range [44]. The normalization process is defined by the following mathematical equation:

(1) $$X_{norm}=\frac{\left(X-X_{min}\right)}{X_{max}-X_{min}}$$

where X is the original signal, $X_{min}$ and $X_{max}$, respectively, represent the minimum and maximum values of the signal. The main reason for choosing min-max normalization is that this method preserves signal morphology and is relatively robust against outliners. In the final stage, signal standardization was performed to address the heterogeneity issue arising from the variable temporal lengths of the PCG recordings. This process involves bringing all signals to a predetermined fixed length in accordance with the requirement of ML algorithms for fixed-sized input vectors [45]. As a result of the standardization process, consistent data structures were obtained, and a homogeneous dataset containing a total of 31,943 data points was created.

**Figure 2:**
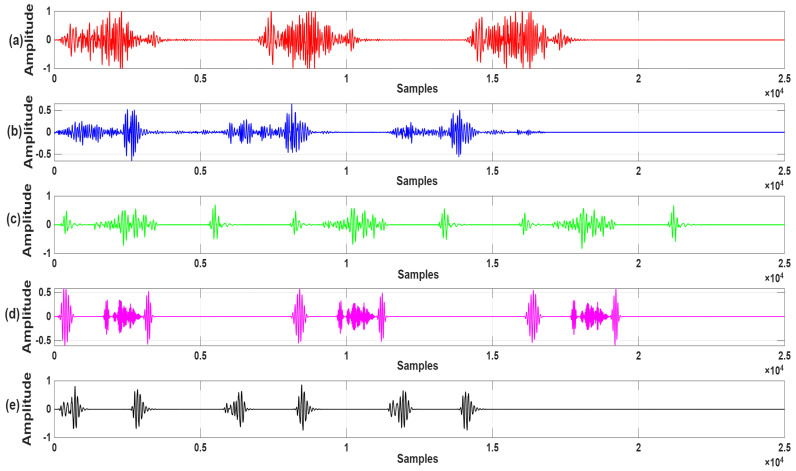
Sample PCG Sensor Signals (**a**) AS, (**b**) MR, (**c**) MS, (**d**) MVP, (**e**) N.

To illustrate the diversity of the dataset and the characteristic features of each pathology class, typical PCG recordings representing five different cardiac conditions are presented in detail in Figure 2. This visual representation contributes to understanding the distinguishing features of different pathologies in terms of signal morphology.

### 2.2. Feature Extraction

In the classification of biomedical signals, the high dimensionality of raw data and the inherent presence of noise represent major challenges that limit the effective and direct learning capabilities of feature extraction-based models. To address these challenges, the feature extraction stage plays a critical role by emphasizing the distinctive characteristics of the signals, enhancing information density, and enabling dimensionality reduction. According to the literature, effective feature extraction not only reduces model complexity and increases computational efficiency but also directly enhances the predictive performance of the classifier [46].

In time series data such as PCG signals, a comprehensive characterization of the signal in the time, frequency, and nonlinear dynamic domains is essential for capturing patterns specific to various cardiac pathologies [47]. In this context, fifteen features were extracted from the time, frequency, and nonlinear dynamic domains to represent the detailed characteristics of PCG signals associated with different cardiac conditions.

Time domain features characterize the fundamental statistical properties of the signal’s amplitude distribution and provide insights into its morphological behavior via parameters such as the mean, standard deviation, skewness, and kurtosis [48]. Frequency domain features, on the other hand, characterize the distribution of signal energy across the frequency spectrum and offer important diagnostic information about pathological murmurs through metrics such as spectral centroid, peak frequency, and bandwidth [49]. Frequency domain features were computed using Fast Fourier Transform (FFT) based spectral analysis. Nonlinear domain features quantify the complexity, irregularity, and memory dynamics of the physiological system generating the heart sound. Entropy based metrics (e.g., permutation entropy, approximate entropy) and parameters such as the Lyapunov exponent are particularly effective in identifying chaotic and nonstationary patterns that traditional linear methods often fail to capture [50]. For nonlinear feature extraction, specific algorithmic parameters were defined to ensure reproducibility. Permutation Entropy was calculated using an embedding dimension of m = 5 and a time delay of τ = 1. Approximate Entropy and Sample Entropy were computed with an embedding dimension of m = 2 and a threshold value of r = 0.2 × std(signal). The Lyapunov Exponent was estimated using an embedding dimension of m = 3 and a time delay of τ = 1. In addition, the Hurst parameter was estimated using the rescaled range (R/S) analysis method.

This multidimensional analysis provides complementary insights into different behavioral aspects of the signal. All computed features were integrated into a unified feature matrix. From a physiological perspective, not all extracted features contribute equally to the characterization of cardiac abnormalities. Some features may contain redundant or less informative patterns that can mask clinically relevant signaling characteristics. Therefore, feature selection plays a crucial role in identifying the most distinctive features associated with pathological heart sound dynamics, improving both classification performance and biomedical interpretability. The complete list of extracted features, their definitions, and mathematical formulations is provided in Table 1.

#### 2.2.1. Feature Selection with Filter Methods

The mRMR method, proposed by Ding and Peng in 2005, is a filter-based, supervised feature selection method [51]. This method aims to minimize the internal correlation and redundancy among the selected features while maximizing the relevance of each feature to the target class. Thus, the aim is to obtain the most informative feature subset that directly contributes to classification performance but does not duplicate each other. mRMR can work effectively with diverse datasets, nonlinear relationships, and multidimensional feature structures by using mutual information as a fitness criterion. The main advantage of the method is that it is computationally efficient, even on high-dimensional datasets, and operates independently of the learning algorithm. In this study, the pseudocode for the features selected by the mRMR method is presented in Algorithm 1.
**Algorithm 1** Feature Selection using mRMR**Require:** Feature set *F*, class labels *C* **Ensure:** Selected feature subset *S* 1: Initialize selected feature subset *S* ← ∅ 2: Compute mutual information between each feature *f* ∈ *F* and class labels *C* 3: **while** stopping criterion is not satisfied do 4:          **for all**
*f* ∈ *F*⧵*S*
**do** 5:                 Compute relevance between *f* and *C* 6:                 Compute redundancy between *f* and features in *S* 7:                 Compute mRMR score: Score(*f*) = Relevance(*f*) − Redundancy(*f*) 8:          **end for** 9:          Add feature with the highest mRMR score to *S* 10: **end while** 11: **return**
*S*

ReliefF is an extension of the original Relief algorithm, preserving the basic mechanism while offering significant improvements [52]. While the original Relief algorithm works by randomly selecting a sample from the dataset and finding the closest neighbors of the same class and different classes, ReliefF has been structured to address multi-class problems by extending this approach. Furthermore, its higher robustness to missing and noisy data contributes to the method producing more reliable results in real-world applications.

The key advantages of ReliefF include universal applicability, low error rate, its ability to account for interactions between features, and its ability to capture local dependencies that other methods may overlook. The fundamental philosophy of the method is based on identifying features that can effectively distinguish examples from different classes. Feature weights, updated at the end of each iteration, reflect the contribution of the relevant feature to classification performance, and features with higher weights are ultimately included in the preferred subset. In this study, ReliefF based feature selection is presented in detail in Algorithm 2.
**Algorithm 2** Feature Selection using ReliefF**Require:** Dataset $D={\left\{\left(x^{\left(i\right)},y^{i}\right)\right\}}_{i=1}^{N}$, number of samples *M*, number of neighbors *k* **Ensure:** Selected feature subset *S* 1: Initialize feature weight vector ***W* ← **0**** 2: **for**
*m* = 1 to *M*
**do** 3:       Randomly select an instance *x* from *D* 4:       Find *k* nearest hits (same class) of *x* 5:       Find *k* nearest misses (different class) of *x* 6:       **for all** features *f* ∈ *F*
**do** 7:             Update *W* (*f*) based on distances 8:        **end for** 9:   **end for** 10: Rank features in descending order of *W* 11: Select top-ranked features to form *S* 12: **return**
*S*

The Kruskal–Wallis test is a supervised, univariate, nonparametric feature selection method used to assess whether two or more independent classes have the same median [53]. This test quantitatively reveals the level of discrimination for each feature based on a comparison of the distributions between classes. The main advantages of the method are its low computational cost, ease of application, and compatibility with a wide range of data types. It enables cost-effective feature reduction, especially in high-dimensional datasets.

The Kruskal–Wallis test calculates a test statistic for each feature, and the significance level is determined by comparing this statistic with the critical value. Values close to zero indicate that the feature has high class discrimination power, meaning that it contributes to classification performance. Thus, features that provide significant discrimination are effectively selected, while those with low discrimination power are eliminated. Pseudo code for this method is given in Algorithm 3.
**Algorithm 3** Feature Selection using Kruskal–Wallis**Require:** Feature set *F*, class labels *C*, significance level *α* **Ensure:** Selected feature subset *S* 1:  **for all** features *f* ∈ *F*
**do** 2:        Perform Kruskal–Wallis *H*-test across class groups 3:        Obtain *p*-value as the significance score of *f* 4:   **end for** 5:   Rank features in ascending order of *p*-value 6:   Select statistically significant features where *p* < *α* 7:   Set *S* as the selected feature subset 8:   **return**
*S*

#### 2.2.2. Feature Selection with Meta-Heuristic Methods

PSO is a heuristic optimization method developed by Kennedy and Eberhart in 1995, inspired by the collective motion and social interaction dynamics of biological communities such as bird flocks or fish schools [54]. In the algorithm, the population consists of individuals called particles, and each particle represents a possible solution in the solution space. The basic principle of PSO is that particles update their positions using both their own best experience (personal best—*pBest*) and the best experience within the entire swarm (global best—*gBest*), converging to the optimal solution over time.

In this stochastic process, particles navigate the solution space by updating their speed and position information over specific iterations. Thus, with each new iteration, they tend to achieve better fitness values (fitness) than previous positions. The search process terminates when the global optimum is reached or when stopping criteria are met. In the context of feature selection, PSO is adapted to identify the optimal feature subset that maximizes the fitness function (e.g., classification accuracy). To this end, each particle is represented by a binary-coded feature selection mask, and each update determines which features to include or exclude. Thus, PSO efficiently explores the search space and arrives at a feature set of optimal or near-optimal size and high discriminatory power. The stages of feature selection using the heuristic PSO are presented in Algorithm 4. For the PSO algorithm, the number of particles and maximum iteration number were defined as 15 and 100, respectively. The cognitive and social coefficients were selected as c_1_ = 2 and c_2_ = 2, while the inertia weight values were linearly varied between W_max_ = 0.9 and W_min_ = 0.4 during the optimization process.
**Algorithm 4** Feature Selection using PSO**Require:** Feature set *F*, population size *Np*, maximum iterations *T*, inertia weight *w*, acceleration coefficients $c_{1},c_{2}$ **Ensure:** Optimal feature subset $S^{*}$ 1:   Initialize particle positions and velocities randomly 2:   Initialize personal best $(p_{Best}$) and global best ($g_{Best}$)  3:   while stopping criterion is not satisfied do 4:         **for all** particles $i=1,\ldots ,N_{p}$ **do** 5:              Evaluate fitness of particle *i* using classification performance 6:              Update velocity:                  $v_{i}\leftarrow wv_{i}+c_{1}r_{1}(p_{Besti}-x_{i})+c_{2}r_{2}(g_{Best}-x_{i})$ 7:              Update particle position: $x_{i}\leftarrow x_{i}+v_{i}$ 8:         **end for** 9:          Update $p_{Best}$ and $g_{Best}$ based on fitness values 10: end while 11: Set *S*∗ $\leftarrow g_{Best}$ 12: **return**
*S*∗

ACO is a heuristic method inspired by the foraging and intra-colony cooperative behaviors of ants, proposed by Dorigo in 1991, and considered one of the most successful swarm intelligence-based optimization algorithms [55]. ACO mimics the way ants follow pheromone trails to find the shortest and most efficient routes between the nest and a food source. In real life, as ants use a particular path, the pheromone concentration on that path increases, increasing the likelihood that other ants will follow the same route. Therefore, over time, the colony collectively converges to the optimal solution by exploring the path with the highest pheromone concentration.

This natural selection and signal amplification mechanism of the algorithm offers robust global optimization capabilities in complex search spaces, and it has been successfully applied to numerous ML and optimization problems in the literature. ACO is a method that has proven particularly effective in feature selection. In this context, each ant creates a subset of candidate features; the more ants select a feature, the higher its level of discrimination. At the end of the process, such a feature will have a higher pheromone concentration than others, increasing the likelihood that other ants within the colony will select that feature. Through this mechanism, ACO iteratively updates pheromone levels to encourage the preference of features that contribute to higher fitness values, resulting in a subset of features that are optimal or convergent over time.

The processing steps for the feature subsets generated by ACO are summarized in Algorithm 5. The subset providing the highest fitness value is determined as the output of the algorithm and represents the best feature combination. For the ACO algorithm, the number of ants and maximum iteration number were set to 10 and 100, respectively. The algorithm parameters were selected as *α* = 1, *β* = 1, pheromone evaporation coefficient *ρ* = 0.2, and φ = 0.5.
**Algorithm 5** Feature Selection using ACO**Require:** Feature set *F*, number of ants *N_a_*, maximum iterations *T*, pheromone parameters *α*, *β*, evaporation rate *ρ* **Ensure:** Optimal feature subset *S*^∗^ 1:  Initialize pheromone matrix *τ* for all features 2:  Initialize heuristic information *η* 3:  **while** stopping criterion is not satisfied **do** 4:        **for all** ants *k* = 1,…, *N_a_* **do** 5:              Construct a feature subset based on pheromone levels (exploitation) and heuristic information (exploration) 6:              Evaluate fitness of the constructed subset using classification performance 7:      **end for** 8:     Update pheromone levels: 9:         Reinforce pheromones on features selected by high-fitness ants 10:       Apply pheromone evaporation with rate *ρ* to avoid stagnation 11: **end while** 12: Select the feature subset with the highest fitness 13: **return**
*S*^∗^

#### 2.2.3. Hybrid Feature Selection Strategy

The hybrid feature selection strategy was utilized in this study as a combination of filter-based classification and meta-heuristic optimization. In this context, first, the 15-dimensional original feature set was evaluated using the mRMR, ReliefF and Kruskal–Wallis methods to extract the most distinctive features. These filtering methods provided a pre-selection stage by reducing low-quality, redundant or statistically less informative features prior to the optimization process. Then, PSO and ACO were applied to the feature subsets extracted from each filtering method, thereby further optimizing the feature combinations. At this stage, each subset was depicted as a binary vector, which encoded the selected and unselected features as 1 and 0, respectively. The quality of each subset was evaluated based on the classification performance achieved with the relevant ML model Thus, the filter stage enabled a rapid and classifier-independent reduction in the feature space, while the meta-heuristic stage provided a more flexible search for more compact and distinct combinations of features. This strategy was used to reduce the computational complexity of PSO and ACO and to obtain feature subsets with better class discrimination capabilities for PCG-based heart disease classification.

### 2.3. ML Algorithms

In this study, three ML algorithms that are widely used in the literature and have different basic working principles were selected for the classification of features obtained from PCG signals: SVM, k-NN, and BT. This diversity aims to increase the robustness of the results by allowing the models to approach the data with different assumptions and approaches.

SVM is a powerful classification algorithm that aims to separate data points belonging to different classes using an optimal hyperplane. The basic principle of the algorithm is to find the hyperplane that maximizes the margin between classes [56,57,58]. For linearly inseparable datasets, kernel functions are used to transform the data into a higher-dimensional feature space, where linear separation is achieved [59]. The main advantages of SVM include its effective performance in high-dimensional spaces, its good generalization ability thanks to margin maximization, and its ability to produce successful results even with small datasets [60]. However, its limitations include long training times for large-scale datasets and the critical importance of kernel function and hyperparameter selection [61].

k-NN is a non-parametric classification algorithm based on the example-based learning paradigm. The algorithm classifies a test example by assigning it to the majority class of its k nearest neighbors in the training set [62]. The fundamental assumption of k-NN is that similar examples are likely to belong to the same class. The main advantages of the algorithm include its simplicity, lack of requirement for a training phase (lazy learning), ability to model nonlinear decision boundaries, and natural ability to work with multi-class problems [63]. On the other hand, high computational cost during the testing phase, sensitivity to the curse of dimensionality, sensitivity to noisy data, and difficulties in determining the optimal k value are among the limitations of the algorithm [64].

BT is an ensemble learning method based on the principle of combining multiple independent decision trees (DTs) generated using the bootstrap sampling technique via ensemble voting or averaging [65]. The main advantage of the method is that it maintains the high interpretability of single DTs while significantly reducing one of their major weaknesses, the tendency to overfitting. The model generates different sub-datasets by applying random resampling (bootstrap) on the training data, and a separate DT is trained on each sub-set; thus, the model instability that arises from small data changes is largely eliminated. BTs are also notable for their ability to easily handle both categorical and continuous variables, being relatively less affected by missing values, and minimizing the impact of outliers on performance. However, due to their ensemble structure, reduced interpretability compared to single DTs and increased computational cost are among the main limitations of the method [66].

### 2.4. Performance Metrics

In classification problems based on ML, the objective evaluation of model performance is of critical importance in terms of demonstrating the effectiveness of the algorithms used and enabling their comparison with each other [67]. In this study, the performance of the models developed for classifying heart sounds derived from PCG signals was comprehensively evaluated using four fundamental metrics based on the confusion matrix. In addition, a 5-fold cross-validation (CV) strategy was employed to ensure a reliable and generalized performance assessment of the proposed models. During the CV process, the dataset was divided into five folds, where four folds were used for training and the remaining fold was used for testing in each iteration. This procedure was repeated five times so that each sample was used once for testing and four times for training. The use of 5-fold CV helps reduce the risk of overfitting and provides a more robust evaluation of model generalization performance. The confusion matrix reveals four main components that form the basis for understanding classifier performance: True Positive (TP—examples that are actually positive and correctly predicted), True Negative (TN—examples that are actually negative and correctly predicted), False Positive (FP—examples that are actually negative but predicted as positive), and False Negative (FN—examples that are actually positive but predicted as negative). The definitions, formulas, and clinical significance of the Accuracy (Acc), Precision (Pre), Recall (Rec), and F1-score (F1) metrics calculated based on these fundamental components are summarized in Table 2.

These metrics provide a comprehensive performance evaluation by summarizing the overall success of the model, as well as detailing the performance variation between classes and the class-specific misclassification rates. This comprehensive evaluation approach allows for the assessment of whether models meet the reliability and robustness requirements necessary for their use in clinical applications.

In addition to these metrics, the Receiver Operating Characteristic (ROC) curve was used in the study. The ROC curve is a key performance indicator that evaluates the success of classification models in distinguishing positive and negative classes. The area under the curve (AUC) summarizes the model’s overall discriminatory power in a single metric, and the closer it is to 1, the higher the classification performance. Therefore, the ROC-AUC value is critical for complementing metrics such as Acc, Pre, and Rec, providing a holistic reflection of the model’s true performance.

## 3. Results

In this study, a comprehensive feature selection and classification analysis was performed to distinguish five cardiac conditions—AS, MR, MS, MVP, and N—using features extracted from PCG signals. The research consists of four parts: (i) Performance analysis of all time, frequency, and nonlinear features; (ii) Performance results obtained with filter-based feature selection methods commonly used in the literature (mRMR, ReliefF, and Kruskal–Wallis); (iii) Performance evaluation based on feature selection performed with PSO and ACO, two meta-heuristic methods frequently preferred in the literature that do not involve biased problems; and (iv) Classification performance analysis of hybrid approaches created by combining filter and meta-heuristic methods. All evaluations were performed using three different ML algorithms—SVM, k-NN, and BT. The entire study was conducted in MATLAB v2020b, and the obtained findings are presented comprehensively in the following sections.

### 3.1. Performance Results Obtained with All Time, Frequency and Nonlinear Features

The average performance values of the five classes, using all time, frequency, and nonlinear features (15 in total), are presented in Table 3.

**Figure 3:**
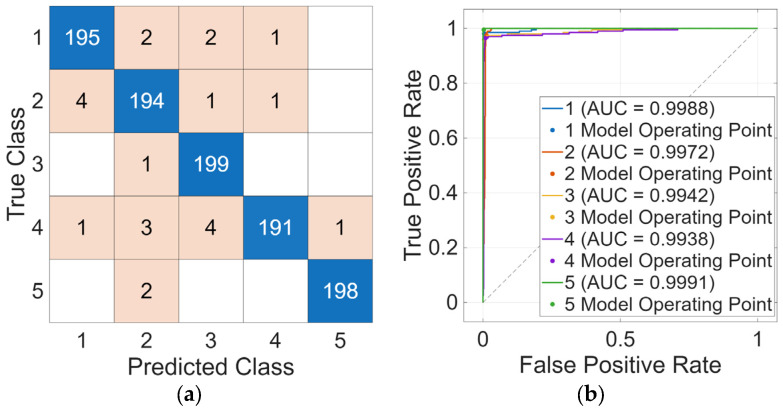
Performance of SVM on all features: (**a**) confusion matrix and (**b**) ROC curve.

As shown by the detailed results in Table 3, the highest performance in the classification analysis was achieved by the SVM algorithm with 99.10% Acc. The confusion matrix and ROC curves for SVM, including five-class cardiac diagnosis, are detailed in Figure 3. The k-NN and BT models produced similar results, both showing 98.40% Acc. Pre, Rec, and F1 score values were also generally high, thus confirming that all features offer strong diagnostic performance in the automatic discrimination of cardiac conditions.

The confusion matrix in Figure 3 shows that the proposed model can distinguish five cardiac pathology classes with high accuracy. Using all features, the model correctly predicted 195, 194, 199, 191, and 198 samples in the 1: AS, 2: MR, 3: MS, 4: MVP, and 5: N classes, respectively, keeping the inter-class error to a minimum. The nearly error-free discrimination, particularly in the MS and N classes, supports the model’s discriminatory power. ROC curves also confirm these findings, with AUC values in the range of 0.9938–0.9991 for all classes. The curves’ close proximity to the graphical area indicates a high true positive rate and a very low false positive rate. Overall, both the confusion matrix and ROC analyses demonstrate that the model offers high accuracy, strong discrimination, and reliable diagnostic performance in the automated diagnosis of cardiac pathologies.

### 3.2. Performance Results of Features Selected with the Filter Method

Filtering methods were used to improve the results obtained from all features and achieve higher performance values. The performance values obtained through this feature selection process, performed using three different methods (mRMR, ReliefF, and Kruskal–Wallis), are presented comprehensively in Table 4.

**Figure 4:**
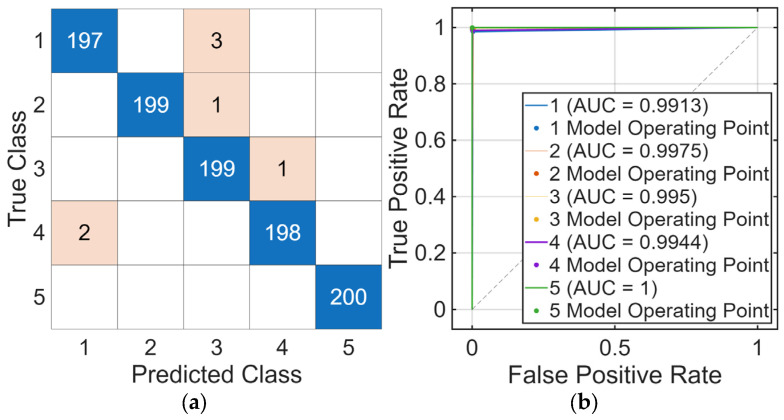
Performance of k-NN on features selected with ReliefF: (**a**) confusion matrix and (**b**) ROC curve.

As can be observed from Table 4, the impact of three different ML algorithms (SVM, k-NN, and BT) and feature selection methods based on mRMR, ReliefF, and Kruskal–Wallis on classification performance has been comprehensively evaluated. When the mRMR method was used, the SVM algorithm achieved an Acc value of 98.80%, while the k-NN and BT algorithms achieved Acc values of 98.20% and 97.80%, respectively. These results show that mRMR selection provides high but similar performance across the three classifiers. The ReliefF method stood out significantly in terms of performance and produced the best results, particularly with the k-NN algorithm, achieving 99.80% Acc, 99.50% Pre, and 99.50% Rec. The ReliefF-SVM combination also demonstrated high diagnostic success with an Acc value of 99.60%. These results show that ReliefF improves the decision boundary separation, thereby enhancing the algorithms’ ability to detect positive classes. When evaluating Kruskal–Wallis-based feature selection, the k-NN model also yielded 99.80% Acc, which is within the standard deviation range of the ReliefF+k-NN (99.80 ± 0.18 vs. 99.80 ± 0.16). The confusion matrix and ROC curve for the ReliefF-k-NN combination are shown in Figure 4. Similarly, the Kruskal–Wallis–SVM combination achieved an Acc value of 99.40%. Although the BT algorithm performed well in all three selection methods, its Acc rates remained lower than those of SVM and k-NN. Overall, the results demonstrate that the feature selection strategy directly impacts classification success, particularly highlighting that ReliefF and Kruskal–Wallis methods, when used in conjunction with SVM and k-NN models, maximize diagnostic performance. The high Acc, Pre, Rec, and F1 scores obtained demonstrate that the developed system can be reliably and effectively used in clinical classification and decision support processes.

A joint analysis of the confusion matrix and ROC curve presented in Figure 4 demonstrates the strong classification capability of the proposed framework. The performance outputs of the k-NN classifier trained on the feature set obtained using the ReliefF feature selection method indicate that the model can distinguish between the five cardiac classes with high accuracy. The Confusion Matrix (Figure 4a) confirms the model’s strong generalization capacity, with the highest Acc achieved in the N class (200/200), where no misclassifications were observed. Errors for other pathological classes remained quite limited; misclassifications were largely concentrated around Class 4 mixing with Class 1 (2 examples) and Class 1 and Class 2 mixing with Class 3 (3 and 1 examples, respectively). ROC curve analysis (Figure 4b) also supports these findings, with AUC values ranging from 0.9913 to 1.0 for all classes, indicating that the model has extremely high discriminatory power. In particular, obtaining AUC = 1.0 for the N class strongly demonstrates that the system can completely distinguish physiological conditions from pathological cardiac disorders. Overall, these results scientifically confirm that the features selected with ReliefF carry a high level of discriminative power in the cardiac classification problem and that the k-NN-based approach provides a reliable, stable, and clinically applicable diagnostic system suitable for decision support processes.

### 3.3. Performance Results of Features Selected with Meta-Heuristic Methods

In this section, the performance effects of feature sets selected using PSO and ACO meta-heuristic methods on SVM, k-NN, and BT algorithms used in the cardiac condition classification problem were examined. The findings show that both feature selection methods provide high classification accuracy (Table 5).

**Figure 5:**
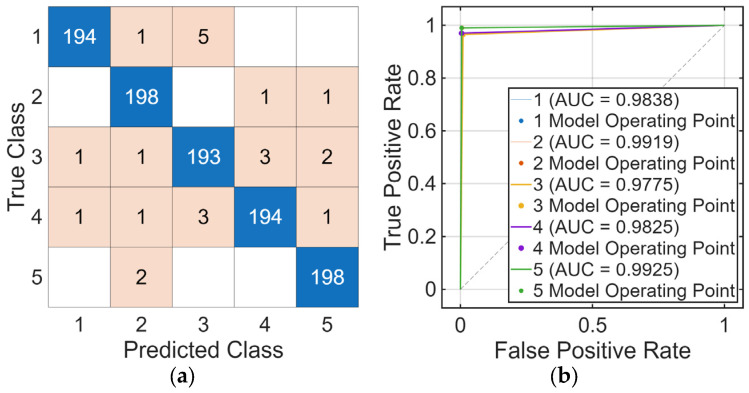
K-NN performance with features selected with ACO: (**a**) confusion matrix and (**b**) ROC curve.

The results presented in Table 5 clearly indicate that k-NN achieved the most stable and superior classification performance among all classifiers when evaluated on the feature subsets generated using PSO and ACO based optimization. Both the PSO+k-NN and ACO+k-NN combinations shared the best performance with 99.00% Acc and 97.50% F1 scores. These results indicate that k-NN most efficiently utilizes the representational power of the selected features. The corresponding confusion matrix and ROC curve details are provided in Figure 5. On the other hand, it has been determined that ACO feature selection is more effective than PSO in improving the performance of other classifiers such as SVM and BT. SVM on the feature set selected by ACO achieved the same excellent results as k-NN, increasing the Acc value from 98.30% in the PSO scenario to 99.00%. This finding reveals that ACO is more successful in determining the feature subset that creates a stronger classification surface for SVM. Overall, in the problem of classifying cardiac conditions, while all feature selection methods provided high performance, it was concluded that the feature sets optimized by ReliefF and ACO, when combined with algorithms such as k-NN and SVM, respectively, provided the most reliable diagnostic results with Acc values reaching 99%.

As illustrated in Figure 5, it is evident that: The k-NN classifier trained on the feature set determined by ACO has demonstrated remarkable diagnostic accuracy in distinguishing cardiac pathologies. The confusion matrix (Figure 5a) reveals that the model has strong generalization performance, with high correct classification values (194, 198, 193, 194, and 198) located on the main diagonal of the model. Upon examining the error distribution, it was observed that misclassifications were quite limited and the most prominent confusion centered around True Class 1 examples being predicted as Class 3. The model’s discriminatory diagnostic power has also been confirmed by ROC curve analyses (Figure 5b). The AUC values obtained for all classes in the range of 0.9775–0.9925 indicate that the system operates with high accuracy, sensitivity, and specificity. In particular, the AUC = 0.9925 recorded for the N class proves that the model has an extremely superior performance in distinguishing healthy individuals from pathological conditions. Additionally, the fact that all operating points are located close to the upper left region of the ROC curves supports that the FP and FN rates remain at minimal levels. In general, these findings scientifically confirm that ACO-based feature selection provides highly discriminative information in the classification of cardiac sound signals and, when used in conjunction with the k-NN algorithm, establishes a reliable, stable, and clinically applicable diagnostic infrastructure for decision support mechanisms.

### 3.4. Performance Results of Designed Hybrid Methods and Selected Features

In this final section, feature subsets were determined using a hybrid model obtained by applying three different filter feature selection methods (mRMR, ReliefF, and Kruskal–Wallis) and meta-heuristic methods (PSO, ACO). The performance of these selected features is compared in Table 6 and Table 7.

**Figure 6:**
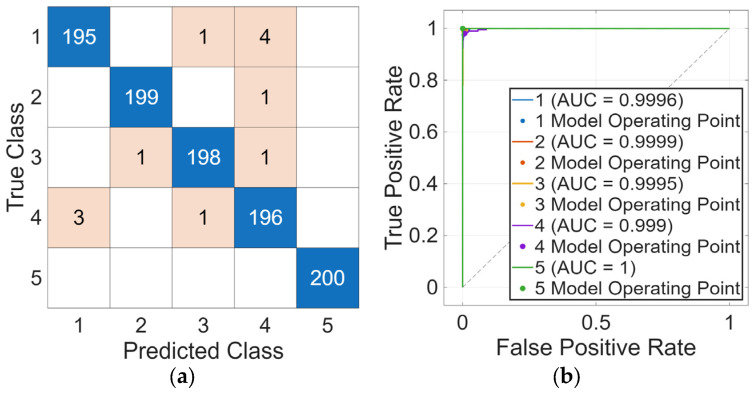
SVM performance with selected features using Kruskal–Wallis+PSO hybrid model: (**a**) confusion matrix and (**b**) ROC curve.

As shown by the results in Table 6, it is evident that the Kruskal–Wallis+PSO combination is the most superior approach in terms of classification success. Specifically, when the SVM and k-NN algorithms were used together with this feature set, they achieved nearly flawless classification with only two FP and two FN, yielding 99.60% Acc and 99.00% F1 scores. This value remains within one standard deviation of the best-performing filter-only combinations (e.g., ReliefF+k-NN: 99.80 ± 0.18), suggesting comparable diagnostic utility. The confusion matrix and ROC curve for the Kruskal–Wallis+PSO+SVM combination, which had the highest success rate, are presented in Figure 6. When examining general performance trends, it was determined that the k-NN algorithm showed the most stable and consistent performance in all feature selection scenarios (99.20–99.60% Acc). However, the BT algorithm lagged behind other classifiers in all combinations; the SVM model paired with mRMR + PSO showed the lowest performance with an Acc value of 98.20%. These results confirm that Kruskal–Wallis-based feature selection provides the most selective and noise-free feature subset for distinguishing cardiac conditions and creates the most diagnostically efficient information space for ML models.

The combination of the hybrid model created using Kruskal–Wallis-based feature selection and PSO with the SVM classifier demonstrated excellent success in classifying cardiac conditions, as shown in Figure 6. The confusion matrix (Figure 6a) shows that the model exhibited extremely high generalization ability across all five classes. With all Class 5 (N) examples (200/200) classified without error and 199 of the Class 2 examples correctly predicted, misclassification rates were kept to a minimum. The most prominent error pattern was concentrated in the misclassification of True Class 4 examples as Class 1 (3 examples). This high accuracy is scientifically supported by ROC Curve (Figure 6b) analysis; AUC values for all classes range from 0.999 to 1.0, proving that the SVM model has near-perfect discrimination power. In particular, the AUC = 1.0 value obtained for Class 5 and the clustering of all classes’ operating points in the upper left corner of the graph confirm that the Kruskal–Wallis+PSO hybrid feature selection provides the most reliable and effective solution for this classification problem.

In the continuation of the study, the performance metrics obtained using the attributes selected by ACO with three different filter attribute selection methods (mRMR, ReliefF, and Kruskal–Wallis) were analyzed. All findings are presented in Table 7.

**Figure 7:**
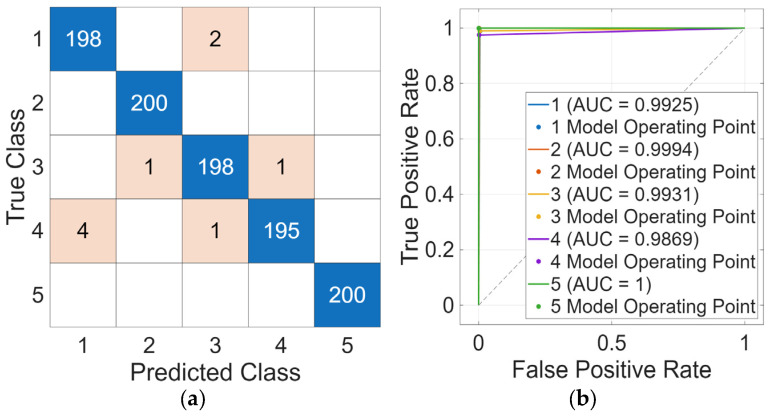
Performance of k-NN with features selected by Kruskal–Wallis+ACO hybrid model: (**a**) confusion matrix and (**b**) ROC curve.

Table 7 clearly shows that the feature selection scenarios where ACO was applied with ReliefF, Kruskal–Wallis, and mRMR preprocessors generally produced high-performance classification results. The highest performance of the study was achieved by the k-NN algorithm with ReliefF+ACO and Kruskal–Wallis+ACO combinations, with Acc values of 99.60% and F1 values of 99.00%. These results confirm that k-NN is the most consistent and powerful classifier compared to all algorithms examined. Within this framework, the detailed confusion matrix and ROC curve for the Kruskal–Wallis+ACO+k-NN combination are shown in Figure 7. In contrast, the mRMR+ACO combination has the lowest performance values. ACO significantly maximized classification performance independently of feature selection methods; particularly when combined with ReliefF and Kruskal–Wallis, it provided nearly flawless separation capability with two (FP) and two (FN) errors. These findings demonstrate that the ReliefF+ACO and Kruskal–Wallis+ACO hybrid approaches provide the most reliable and effective feature sets for classifying cardiac conditions.

Figure 7 clearly demonstrates that, the combination of the hybrid model created using Kruskal–Wallis-based feature selection and ACO with the SVM classifier has demonstrated extremely high performance in classifying cardiac conditions. The Confusion Matrix (Figure 7a) clearly shows that the model has strong generalization capabilities across all five classes. In particular, the error-free classification of all Class 2 and Class 5 examples (200/200) proves the model’s flawless sensitivity and specificity in distinguishing these classes. However, matrix analysis revealed that the most prominent errors were concentrated in the assignment of True Class 4 examples to Class 1 (4 examples) and True Class 1 examples to Class 3 (2 examples).

This high accuracy in classification performance is also supported by the ROC curve (Figure 7b). The fact that the AUC values for all classes range from 0.9869 to 1.0 clearly demonstrates the superior discrimination power of the SVM model. Specifically, the AUC = 1.0 value obtained for Class 5 and the positioning of all operating points near the upper left corner of the graph indicate both that the model’s sensitivity-specificity balance is at an optimal level and that the Kruskal–Wallis+ACO hybrid feature selection offers the most reliable and effective solution for this classification problem.

**Figure 8:**
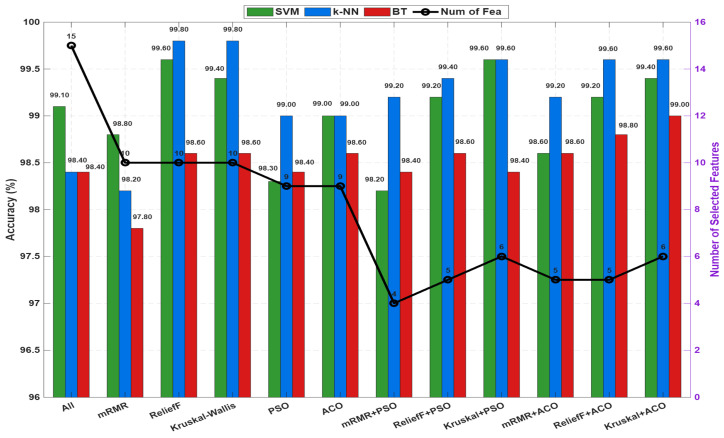
Accuracy performance results of algorithms against selected features.

Detailed graphs of all these results, both in terms of methods and number of features, are provided in Figure 8.

The comparative analysis presented in Figure 8 comprehensively demonstrates the impact of different feature selection approaches on classification performance. When the Acc results of three different ML algorithms (SVM, k-NN, and BTs) are evaluated together, it is clear that feature selection directly impacts model performance. Although high accuracy is achieved when all features are used, alternative feature subsets generated by some filters and meta-heuristics methods achieve similar or even higher accuracy levels with fewer dimensions. In particular, the combinations of ReliefF, Kruskal–Wallis, ReliefF+PSO, Kruskal–Wallis+PSO, ReliefF+ACO, and Kruskal–Wallis+ACO outperformed the full-feature model in terms of average Acc, with ReliefF and Kruskal–Wallis+ACO standing out as the most consistent approaches.

**Figure 9:**
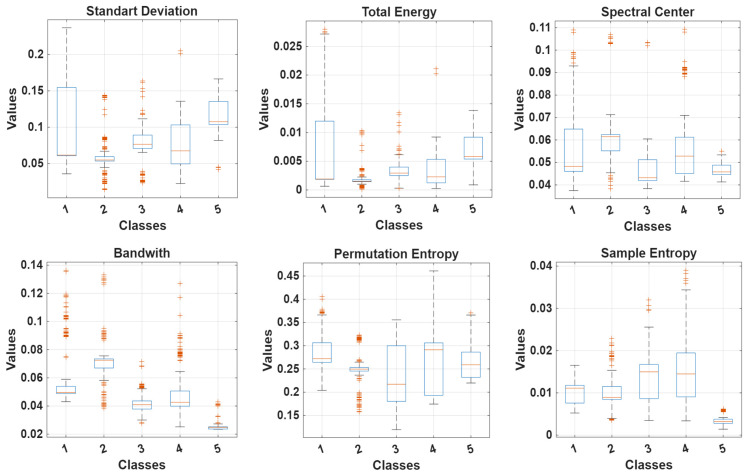
Boxplot display of features with high classification performance.

At the same time, when examining the number of selected features shown on the graph, it is seen that the relationship between the decrease in feature size and Acc is not linear, but that optimal feature subsets enhance learning performance without adding extra information load to the model. In particular, hybrid methods containing only 5 or 6 attributes have been observed to provide equivalent or even higher accuracy in some cases compared to the full model with 15 attributes. This confirms that effective attribute selection not only reduces computational cost, but also improves classification performance. The boxplot representation of the 6 attributes with the highest discriminative power is shown in Figure 9.

The boxplots of the features shown in Figure 9 clearly demonstrate that there are significant distribution differences between cardiac classes and that these features contribute significantly to the success of the classification model. The Standard Deviation feature exhibited a wider distribution, particularly in Class 1 samples, while showing more compact values in Class 2 and Class 3, proving that variance-based separation is effective between classes. In terms of the Total Energy characteristic, Class 1 having distinctly high values supported the relationship between the pathological condition and energy density. The Spectral Center and Bandwidth features provided important discriminating clues in the spectral dimension; Classes 2 and 4 showing higher center frequency and bandwidth distributions demonstrated that pathological conditions could be clearly distinguished on the phonocardiographic spectrum. The other two complexity-based features, Permutation Entropy and Sample Entropy, were characterized by the lowest entropy values, particularly in Class 5 individuals; conversely, higher complexity levels were observed in pathological classes. This situation confirms that cardiac anomalies disrupt signal regularity and that irregularity coefficients function as powerful biomarkers for diagnosis. Overall, boxplot analyzes scientifically support that each feature set provides clear distinctions between classes and that these selected features are the key determinants of high classification performance.

Overall, the study findings clearly demonstrate that achieving an optimal balance between high Acc, low dimensionality, and strong generalizability is possible through appropriate feature selection strategies. Thus, the approach developed for PCG-based cardiac diagnosis systems offers a robust solution in terms of algorithmic efficiency and medical interpretability.

## 4. Discussion

In this study, a comprehensive and multi-stage feature selection–modeling strategy was adopted for the classification of cardiac conditions. In the first stage, filter-based methods such as mRMR, ReliefF, and Kruskal–Wallis were used to identify the statistically most discriminative features. Subsequently, meta-heuristic algorithms such as PSO and ACO were employed to achieve a more optimized and exploratory selection in the solution space, thereby reducing the meaningful features identified by the filter methods into smaller and more effective subsets. Furthermore, the hybrid combination of filtering and meta-heuristic strategies contributed to obtaining stronger, more balanced, and more generalizable models compared to single-stage feature selection. In the final stage, SVM, k-NN, and BTs classifiers were applied with the selected feature sets, and the contribution of each method to the model was evaluated comprehensively through Acc, Pre, Rec and F1 scores, confusion matrices, and ROC-AUC results. The findings clearly demonstrate that the proposed feature selection and classification framework provides high diagnostic performance.

The hybrid feature selection framework combines the complementary strengths of filter-based and meta-heuristic optimization approaches. Filter methods such as mRMR, ReliefF, and Kruskal–Wallis provide a fast and computationally efficient pre-liminary screening stage by ranking or selecting features according to statistical relevance, redundancy reduction, or local discriminative ability. However, filter-based methods do not necessarily optimize the final feature subset with respect to the classifier-dependent objective function and may therefore overlook feature combinations that jointly improve classification performance. In contrast, meta-heuristic algorithms such as PSO and ACO can explore the feature-subset search space more flexibly and identify near-optimal feature combinations, although this usually increases computational cost. Therefore, the proposed hybrid strategy first reduces the original feature space through filter-based pre-selection and then refines the candidate subsets using meta-heuristic optimization. This two-stage structure reduces the search burden of PSO and ACO, limits feature redundancy, and improves the discriminative capacity of the selected subset compared with standalone feature selection strategies (e.g., using only filter methods or only meta-heuristic algorithms).

To the best of our knowledge, existing studies on PCG-based cardiac classification have not systematically combined filter-based methods with meta-heuristic optimization within a hybrid framework; most employ either filter or meta-heuristic methods alone. Our hybrid results consistently outperform or match those of standalone methods on the same dataset while using significantly fewer features (Table 4, Table 5, Table 6 and Table 7).

The literature comparison summarized in Table 8 shows that high Acc rates have been reported for cardiac disease classification based on PCG signals using different methods; however, there are significant performance differences between studies. For example, although the multi-feature-based deep neural network (DNN) model proposed by [24] achieves 97.00% Acc, the Rec value remaining at 94.50% indicates weakness in interclass discriminability. Similarly, although the Wavenet-based model [20] produced 97.00% Acc, the drop in recall to 92.50% negatively affected the classification balance. More advanced approaches have increased Acc values, with the TCN-MoE and community-based method [37] delivering one of the strongest results in the literature with 98.80% Acc and a 99.40% F1 score. The AOCT-I/AOCT-II-based model [36] also achieved high-level performance with 99.00% Acc. Although the codec development-based analysis [18] achieved 98.00% Acc and balanced metrics, it did not provide as clear discrimination as DL-based models.

Compared to these studies, the performance results obtained in this study demonstrate that the proposed methods show competitive or superior performance to many existing approaches in terms of Acc and consistency. Specifically, the Kruskal–Wallis+k-NN and ReliefF+k-NN combinations achieved Acc and F1 scores comparable to or higher than DL-based models, achieving 99.80% Acc and 99.50% F1 score. The PSO+k-NN and ACO+k-NN combinations, which use only meta-heuristic algorithms, also produced results very close to high-performing DL methods such as AOCT and TCN-MoE, with Acc values of 99.00%, respectively. Furthermore, the Kruskal–Wallis+PSO+k-NN and Kruskal–Wallis+ACO+k-NN hybrid structures demonstrated performance equivalent to the strongest models in the literature, with 99.60% Acc and 99.00% F1 score. These findings demonstrate that when filter methods identify the most meaningful features and meta-heuristic algorithms combine their ability to search for optimal feature sets, it is possible to develop systems that are as powerful as complex DL architectures while relying on a lower-dimensional feature representation and simpler conventional classifiers.

The results obtained demonstrate that achieving high performance in PCG-based diagnostic systems is not solely dependent on the complexity of DL-based models; rather, appropriate feature selection strategies and classifier combinations significantly influence diagnostic success. In this context, the proposed hybrid approach offers a valuable alternative to strong models in the literature not only in terms of Acc but also in terms of computational efficiency, model transparency, and clinical applicability.

Overall, the findings indicate that feature selection plays a critical role in maximizing diagnostic accuracy in PCG-based diagnostic systems. While all methods show clinically promising performance, hybridizing filter-based strategies such as ReliefF and Kruskal–Wallis with meta-heuristic algorithms like PSO and ACO produced the most reliable results. This clearly demonstrates the potential of the proposed framework to support clinical decision making processes, reduce subjective assessment based on auscultation, and contribute to the early diagnosis and screening of cardiac valve diseases.

Although the Acc values reported in this study are high, the findings should be interpreted within certain methodological boundaries. All experiments relied on the same 5-fold CV strategy, which offers a useful internal estimate of how well the model generalizes. However, because no independent external test set was used, these results remain tied to the specific dataset and validation protocol employed here.

Moreover, the dataset—obtained from a public GitHub repository and typical of PhysioNet/CinC-based PCG classification studies—provides only limited detail of re-cording conditions, sensor characteristics, patient demographics, and environmental noise. Therefore, the performance obtained reflects controlled data conditions rather than evidence of readiness for clinical use. The proposed framework’s applicability in different devices, recording environments, patient populations, and clinical settings still needs to be validated.

From a clinical engineering perspective, the selected features provide clinically meaningful information about cardiac sound characteristics. Time-domain features reflect amplitude-related morphological variations, frequency-domain features capture spectral changes associated with pathological heart sounds, and nonlinear features quantify the complexity and irregularity of PCG signals. Therefore, the selected feature subset is not only statistically discriminative but also physiologically interpretable. The computational efficiency of the method stems from this reduced feature set (only 5–6 features) and the use of simple traditional ML classifiers. From an algorithmic complexity perspective, k-NN requires only a few thousand distance calculations per inference, and SVM relies on a small number of support vectors, resulting in a very low computational burden. Such operations are generally considered feasible for real-time implementation on low-power embedded platforms commonly used in wearable devices, such as intelligent stethoscopes or low-power screening devices. In a practical deployment scenario, feature selection would be performed offline, whereas only preprocessing, feature extraction, and classifier inference would be executed online. No direct hardware-matched comparison with DL-based models was carried out, and precise inference latency, memory footprint, and power consumption depend on the specific hardware and software environment. Therefore, direct validation on target embedded platforms remains an important direction for future studies.

It should also be noted that all performance comparisons between different feature selection methods are based on mean Acc and standard deviations from 5-fold CV. Formal statistical testing (e.g., McNemar’s test) was not performed to compare each pair of methods, as the primary goal was to demonstrate the feasibility of hybrid feature selection rather than to establish superiority of one specific combination. The overlapping standard deviations (e.g., ReliefF+k-NN: 99.80 ± 0.18; Kruskal–Wallis+PSO+k-NN: 99.60 ± 0.32) suggest that the top-performing models (99.60–99.80% Acc) are practically equivalent within the variability of the dataset.

## 5. Conclusions

This study proposes a multi-stage and systematic feature selection–modeling approach to achieve high diagnostic accuracy in cardiac condition classification based on PCG signals. The use of filter-based (Kruskal–Wallis and ReliefF) and meta-heuristic (PSO and ACO) methods, both separately and in a hybrid form, has enabled the more conscious, optimized, and generalizable selection of feature subsets. The evaluation of different classifiers, primarily k-NN and SVM, with the selected feature sets revealed that the proposed framework provides highly reliable performance at a clinical level for PCG-based diagnostic systems.

Although previous studies in the literature have reported that DL-based models achieve high Acc rates, this study has demonstrated that the combined use of filtering and meta-heuristic methods can produce results that rival deep models and even outperform them in some cases. Specifically, the Kruskal–Wallis+k-NN and ReliefF+k-NN combinations achieved Acc values (99.80%) that are among the highest reported for PCG-based cardiac classification using conventional classifiers, and are competitive with recent DL-based studies, with 99.80% Acc and 99.50% F1 score. Furthermore, the Kruskal–Wallis+PSO+k-NN and Kruskal–Wallis+ACO+k-NN hybrid structures also offered comparable success to complex DL-based models, with 99.60% Acc and 99.00% F1 score. This finding demonstrates that heavy and computationally costly architectures are not necessarily required to achieve high accuracy in PCG signals.

The computational advantage of the proposed method is rooted in its feature-based design. Rather than feeding raw PCG signals to the model, specific handcrafted features were extracted and used with traditional ML classifiers. This keeps the feature space small and the model structure simple. No direct quantitative comparison against CNN-based models was conducted under identical hardware conditions. The computational advantage of the proposed method is therefore derived from the reduced feature dimension and the simplicity of the conventional classifiers, rather than from empirical runtime measurements. Nonetheless, the low algorithmic complexity suggests that real-time implementation is plausible, and future work will include direct hardware benchmarking.

The results obtained show that properly designed feature selection strategies significantly affect classification performance, while optimization and hybridization steps greatly increase stability, generalizability, and fault tolerance. In this regard, the proposed method offers a robust and innovative alternative to the existing literature on automatic diagnosis of cardiac valve diseases, with advantages such as computational efficiency, model simplicity, clinical applicability, and hardware simplicity.

Future work should benchmark the proposed framework against representative CNN and other DL PCG classifiers using the same hardware and experimental setup, reporting training time, inference speed, memory use, model size, parameter counts, and computational cost alongside classification performance, to determine real-world practicality. The method should also be validated on larger, independent datasets covering different recording conditions, noise levels, and patient groups to confirm robustness. Real-time embedded implementations and hybrid models that combine DL with feature selection could be explored. These steps would enable a more realistic assessment of the clinical and screening potential of PCG-based computer-aided diagnosis systems.

## Figures and Tables

**Table 1:** The definition of the used features.

Feature	Analyses Type	Definition	Mathematical Equation
Mean	Time	Arithmetic mean of the data	μ = $\frac{1}{N}$ $\sum_{i:1}^{N}x_{i}$
Standard Deviation	Time	Measures the distribution of the data	σ = $\sqrt{\frac{1}{N}}\sum_{i:1}^{N}{\left(xi-\mu \right)}^{2}$
Skewness	Time	Measures the asymmetry of the data	S = $\frac{1}{N}\sum_{i:1}^{N}{\left(\frac{xi-\mu }{\sigma }\right)}^{3}$
Kurtosis	Time	Measures the peakedness of the data	K = $\frac{1}{N}\sum_{i:1}^{N}{\left(\frac{xi-\mu }{\sigma }\right)}^{4}$ − 3
Range	Time	Difference between the maximum and minimum	R = max(x_i_) − min(x_i_)
Total Energy	Frequency	Total energy of the signal	E = ∑xi2
Average Frequency	Frequency	Weighted average of frequencies	Favg =$\frac{\sum fi*P(fi)}{\sum P(fi)}$
Spectral Center	Frequency	Center of the frequency distribution	SC =$\frac{\sum f.IX(f)I}{\sum IX\left(f\right)I}$
Peak Frequency	Frequency	Frequency with maximum amplitude	Fpeak = argmaxP(fi)
Bandwidth	Frequency	Width of frequency components	B = $\sqrt{\frac{\sum {\left(fi-SC\right)}^{2}*P(fi)}{\sum P(fi)}}$
Lyapunov Exponent	Nonlinear	Chaotic structure of the systems	λ = $\underset{t\to \infty }{\lim}\frac{1}{t}\log\frac{\varphi (t)}{\varphi (0)}$
Hurst Parameter	Nonlinear	Long-term dependence of the signals	Statistical estimation
Permutation Entropy	Nonlinear	Irregularity of the sequence	Algorithmic calculation
Approximate Entropy	Nonlinear	Complexity of the time series	ApEn(m, r) = Φm(r)-Φm+1(r)
Sample Entropy	Nonlinear	Unpredictability of the signals	SampEn = −$\log(\frac{A(m)}{B(m)}$)

**Table 2:** Performance Metrics: Definitions, Formulas and Clinical Significance [56].

Metric	Definition	Formula	Clinical Significance
Acc	Total proportion of correctly classified instances	$\frac{TP+TN}{TP+TN+FP+FN}$	Summarizes the overall classification performance of the model
Pre	Reliability of positive predictions	$\frac{TP}{TP+FP}$	Important in scenarios where false positive predictions are costly.
Rec	Positive case detection rate	$\frac{TP}{TP+FN}$	Critical when missing a diseased case poses a high clinical risk.
F1	Harmonic mean of precision and recall	2 × $\frac{Pre*Rec}{Pre+Rec}$	Provides a balanced performance assessment under class imbalance.

**Table 3:** Average performance metrics for all features.

Algorithms	TP	TN	FP	FN	Acc (%)	Pre (%)	Rec (%)	F1 (%)
SVM	195	796	4	5	99.10 ± 0.18	97.99 ± 0.20	97.50 ± 0.22	97.74 ± 0.21
k-NN	192	792	8	8	98.40 ± 0.26	96.00 ± 0.24	96.00 ± 0.24	96.00 ± 0.24
BT	192	792	8	8	98.40 ± 0.31	96.00 ± 0.35	96.00 ± 0.35	96.00 ± 0.35

**Table 4:** Average performance metrics of features selected with Filter Methods.

**mRMR**	**Algorithms**	**TP**	**TN**	**FP**	**FN**	**Acc (%)**	**Pre (%)**	**Rec (%)**	**F1 (%)**
	SVM	194	794	6	6	98.80 ± 0.15	97.00 ± 0.40	97.00 ± 0.40	97.00 ± 0.40
	k-NN	191	791	9	9	98.20 ± 0.22	95.50 ± 0.46	95.50 ± 0.46	95.50 ± 0.46
	BT	189	789	11	11	97.80 ± 0.28	94.50 ± 0.38	94.50 ± 0.38	94.50 ± 0.38
**ReliefF**	SVM	198	798	2	2	99.60 ± 0.22	99.00 ± 0.38	99.00 ± 0.38	99.00 ± 0.38
	k-NN	199	799	1	1	99.80 ± 0.18	99.50 ± 0.32	99.50 ± 0.32	99.50 ± 0.32
	BT	193	793	7	7	98.60 ± 0.26	96.50 ± 0.38	96.50 ± 0.38	96.50 ± 0.38
**Kruskal–Wallis**	SVM	197	797	3	3	99.40 ± 0.32	98.50 ± 0.40	98.50 ± 0.40	98.50 ± 0.40
	k-NN	199	799	1	1	99.80 ± 0.16	99.50 ± 0.19	99.50 ± 0.19	99.50 ± 0.19
	BT	193	793	7	7	98.60 ± 0.26	96.50 ± 0.21	96.50 ± 0.21	96.50 ± 0.21

**Table 5:** Average performance metrics of features selected by meta-heuristic methods.

**PSO**	**Algorithms**	**TP**	**TN**	**FP**	**FN**	**Acc (%)**	**Pre (%)**	**Rec (%)**	**F1 (%)**
	SVM	192	791	9	8	98.30 ± 0.38	95.52 ± 0.52	96.00 ± 0.48	95.76 ± 0.50
	k-NN	195	795	5	5	99.00 ± 0.42	97.50 ± 0.40	97.50 ± 0.40	97.50 ± 0.40
	BT	192	792	8	8	98.40 ± 0.36	96.00 ± 0.45	96.00 ± 0.45	96.00 ± 0.45
**ACO**	SVM	195	795	5	5	99.00 ± 0.40	97.50 ± 0.36	97.50 ± 0.36	97.50 ± 0.36
	k-NN	195	795	5	5	99.00 ± 0.32	97.50 ± 0.29	97.50 ± 0.29	97.50 ± 0.29
	BT	193	793	7	7	98.60 ± 0.36	96.50 ± 0.45	96.50 ± 0.45	96.50 ± 0.45

**Table 6:** Average performance results of features selected with hybrid methods and PSO Methods.

**mRMR** **+PSO**	**Algorithms**	**TP**	**TN**	**FP**	**FN**	**Acc (%)**	**Pre (%)**	**Rec (%)**	**F1 (%)**
	SVM	191	791	9	9	98.20 ± 0.44	95.50 ± 0.60	95.50 ± 0.60	95.50 ± 0.60
	k-NN	196	796	4	4	99.20 ± 0.38	98.00 ± 0.18	98.00 ± 0.18	98.00 ± 0.18
	BT	192	792	8	8	98.40 ± 0.48	96.00 ± 0.42	96.00 ± 0.42	96.00 ± 0.42
**ReliefF** **+PSO**	SVM	196	796	4	4	99.20 ± 0.50	98.00 ± 0.36	98.00 ± 0.36	98.00 ± 0.36
	k-NN	197	797	3	3	99.40 ± 0.35	98.50 ± 0.42	98.50 ± 0.42	98.50 ± 0.42
	BT	193	793	7	7	98.60 ± 0.60	96.50 ± 0.50	96.50 ± 0.50	96.50 ± 0.50
**Kruskal–Wallis** **+PSO**	SVM	198	798	2	2	99.60 ± 0.25	99.00 ± 0.38	99.00 ± 0.38	99.00 ± 0.38
	k-NN	198	798	2	2	99.60 ± 0.32	99.00 ± 0.20	99.00 ± 0.20	99.00 ± 0.20
	BT	192	792	8	8	98.40 ± 0.36	96.00 ± 0.48	96.00 ± 0.48	96.00 ± 0.48

**Table 7:** Average performance results of features selected with Hybrid Filter and ACO Methods.

**mRMR** **+ACO**	**Algorithms**	**TP**	**TN**	**FP**	**FN**	**Acc (%)**	**Pre (%)**	**Rec (%)**	**F1 (%)**
	SVM	193	793	7	7	98.60 ± 0.28	96.50 ± 0.48	96.50 ± 0.48	96.50 ± 0.48
	k-NN	196	796	4	4	99.20 ± 0.40	98.00 ± 0.45	98.00 ± 0.45	98.00 ± 0.45
	BT	193	793	7	7	98.60 ± 0.52	96.50 ± 0.35	96.50 ± 0.35	96.50 ± 0.35
**ReliefF** **+ACO**	SVM	196	796	4	4	99.20 ± 0.26	98.00 ± 0.28	98.00 ± 0.28	98.00 ± 0.28
	k-NN	198	798	2	2	99.60 ± 0.18	99.00 ± 0.20	99.00 ± 0.20	99.00 ± 0.20
	BT	194	794	6	6	98.80 ± 0.14	97.00 ± 0.18	97.00 ± 0.18	97.00 ± 0.18
**Kruskal–Wallis** **+ACO**	SVM	197	797	3	3	99.40 ± 0.32	98.50 ± 0.26	98.50 ± 0.26	98.50 ± 0.26
	k-NN	198	798	2	2	99.60 ± 0.20	99.00 ± 0.25	99.00 ± 0.25	99.00 ± 0.25
	BT	195	795	5	5	99.00 ± 0.30	97.50 ± 0.40	97.50 ± 0.40	97.50 ± 0.40

**Table 8:** Literature comparison.

Reference	Method	Acc (%)	Pre (%)	Rec (%)	F1 (%)
[24]	DNN, Multiple features	97.00	-	94.50	-
[20]	Wavenet	97.00	-	92.50	-
[37]	TCN-MoE, Ensemble learning	98.80	99.40	99.40	99.40
[36]	AOCT-I, AOCT-II	99.00	99.00	99.00	99.00
[18]	MS, Codec enhancement	98.00	97.90	98.00	-
This study	SVM	99.10	97.99	97.50	97.74
	Kruskal–Wallis+k-NN	99.80	99.50	99.50	99.50
	ReliefF+k-NN	99.80	99.50	99.50	99.50
	PSO+k-NN	99.00	97.50	97.50	97.50
	ACO+k-NN	99.00	97.50	97.50	97.50
	Kruskal–Wallis+ACO+k-NN	99.60	99.00	99.00	99.00
	Kruskal–Wallis+PSO+k-NN	99.60	99.00	99.00	99.00

## Data Availability

The dataset used in this study is publicly available on GitHub at the following repository: Classification-of-Heart-Sound-Signal-Using-Multiple-Features (https://github.com/yaseen21khan/Classification-of-Heart-Sound-Signal-Using-Multiple-Features-/ accessed on 1 March 2024).
